# Evidence summary on the non-pharmacological management of sleep disorders in shift workers

**DOI:** 10.1007/s11325-023-02901-5

**Published:** 2023-08-16

**Authors:** Jin-yu Wu, Hui Li, Jun-kun Shuai, Yue He, Peng-cheng Li

**Affiliations:** 1 Department of Orthopedic Surgery and Orthopedic Research Institute, West China Hospital, Sichuan University, No.37, Guoxue Alley, Chengdu, 610044 Sichuan China; 2 West China School of Nursing, Sichuan University, No.37, Guoxue Alley, Chengdu, 610044 Sichuan China; 3 Department of Emergency Medicine, Affiliated Hospital of Chengdu University, No.82, North 2nd Section, 2nd Ring Road, Chengdu, 610081 Sichuan China

**Keywords:** Shift work, Sleep, Evidence-based nursing, Evidence summary

## Abstract

**Purpose:**

This study aimed to evaluate, and integrate the relevant evidence on the non-pharmacological management of sleep disorders in shift workers to provide a reference for improving sleep of shift workers.

**Methods:**

According to the “6S” pyramid model of evidence, a comprehensive search was conducted in evidence-based databases, including BMJ-Best Practice, UpToDate, DynaMed, Cochrane Library, and Joanna Briggs Institute (JBI); clinical practice guideline websites, such as the Guidelines International Network; professional association websites, such as the World Sleep Society; and literature databases, including PubMed, Embase, CINAHL, China National Knowledge Infrastructure (CNKI), Wanfang Database, and Chinese Biology Medicine disc (CBM) from inception to November 30, 2022. Two researchers independently evaluated the literature in accordance with the evaluation standards; conducted the extraction, classification, and synthesis of the evidence; and evaluated its grade and recommendation grade.

**Results:**

A total of 18 studies were included, including 2 clinical decisions, 2 guidelines, 3 expert consensuses, and 11 systematic reviews. In total, 25 pieces of evidence were summarized from 6 aspects: sleep assessment, sleep scheduling, sleep hygiene, light therapy, workplace intervention, and other managements.

**Conclusion:**

This study summarized the best evidence for the non-pharmacological management of sleep disorders in shift workers. Shift workers should reasonably arrange their sleep time and develop good sleep hygiene. Additionally, work organizations should jointly promote sleep to improve the sleep conditions of shift workers and promote their physical and mental health.

## Introduction

With the increasing demand for round-the-clock work in society, approximately 15 to 30% of full-time workers worldwide are engaged in shift work [1, 2]. The term shift work refers to any work schedule that falls outside the hours of 7 a.m. to 6 p.m., including evening, night, and early morning shifts, as well as fixed or rotating schedules [1]. Shift work has been found to have a negative effect on human health through circadian rhythm disruption, sleep deprivation, and suboptimal health behaviors [3]. Sleep is a fundamental human need and plays a critical role in maintaining human alertness. However, approximately 25 to 30% of shift workers experience insomnia symptoms, and approximately 10% meet the diagnostic criteria for shift work sleep disorder (SWSD). Studies have shown that sleep deprivation and shift work can result in adverse outcomes such as traffic accidents, increased alcohol and drug use, and serious medical errors [4, 5]. In addition, shift work or other non-standard working hours have significant potential to encroach on valuable “social time” and produce “social desynchronization” by reducing the time available for social interaction, leisure, and family activities. This desynchronization of personal and social behavioral structures may increase impairments to social life and affect balance between the work and non-work domains and social participation [6]. Therefore, extensive research on the affecting factors [7], adverse consequences [8], and intervention strategies [9] of shift work or SWSD has been conducted.

However, despite the higher prevalence of sleep disorders among shift workers [10], especially among medical personnel, and the negative effects that they may have, attention to this research field is relatively insufficient in China. Although pharmaceutical intervention can partially address SWSD, it may also cause adverse reactions. Recently, non-pharmacological interventions such as sleep hygiene and light therapy have been increasingly studied [11, 12]. However, the intervention effects have mixed conclusions, and the quality of the studies vary, with insufficient evidence-based support. Therefore, this study aimed to evaluate, and integrate the best evidence for the non-pharmacological management of sleep disorders in shift workers, to provide the relevant knowledge for shift workers and work organizations on SWSD management, to improve the sleep time and quality of shift workers, and to promote their physical and mental health.

## Materials and methods

### Question identification

Based on the clinical question “How to manage non-pharmacological interventions for sleep disorders in shift workers,” we used the Problem Development Tool of the Evidence-based Nursing Center of Fudan University and identified questions according to the PIPOST principle [13]. The initial question was as follows. Population (P): the target population was shift workers; Intervention (I): the intervention method included non-pharmacological management recommendations or interventions for sleep disorders in shift workers, such as sleep scheduling, sleep hygiene, light therapy, and workplace interventions; Professional (P): the evidence could be applied by healthcare professionals, organizational managers, and researchers involved in shift work, as well as interested members of the public; Outcome (O): the outcomes included sleep time, sleep quality, and other relevant outcomes; Setting (S): the evidence could be applied in the workplace or home settings; and Type of evidence (T): the types of evidence included guidelines, expert consensuses, systematic reviews, meta-analyses, evidence summaries, clinical decisions, and best practices.

### Retrieval strategy

According to the “6S” pyramid model of evidence-based resources, a top-down search was conducted on the following websites and databases: (i) Evidence-based databases: BMJ-Best Practice, UpToDate, DynaMed, Cochrane Library, and Joanna Briggs Institute (JBI) Library; (ii) Clinical practice guideline websites: World Health Organization (WHO), Guidelines International Network (GIN), National Institute for Health and Care Excellence (NICE), Scottish Intercollegiate Guidelines Network (SIGN), Canadian Medical Association Clinical Practice Guidelines Infobase (CMA CPG), Registered Nurses Association of Ontario (RNAO), New Zealand Guidelines Group (NZGG), Australian Government National Health and Medical Research Council (NHMRC), and Medlive; (iii) Professional association websites: World Sleep Society, American Academy of Sleep Medicine, National Institute for Occupational Safety and Health, National Sleep Foundation, Australian Sleep Association, Work Time Society, Polish Sleep Research Society, and China Sleep Research Society; (iv) English and Chinese databases: PubMed, Embase, CINAHL, China National Knowledge Infrastructure (CNKI), Wanfang Database, and China Biology Medicine disc (CBM). We used the English search terms “shift work/shift worker*/personnel staffing and scheduling/shift work schedule/rotating shift/night work/night shift/light at night/working shift/night worker*/” and “sleep/insomnia/sleep initiation and maintenance disorders/sleep disorders, circadian rhythm/dyssomnias/jet lag syndrome/sleep arousal disorders/sleep-wake transition disorders/sleep disorder*.” The retrieval time ranged from the database establishment to November 30, 2022.

The search strategy in CINAHL was as follows: (((MH “Shiftwork”) OR (MH “Shift Workers”) OR (MH “Personnel Staffing and Scheduling+”)) OR AB Shift Work Schedule OR AB shift work OR AB shift-work OR AB shift worker* OR AB rotating shift OR AB night work OR AB night shift OR AB light at night OR AB working shift OR AB night worker*) AND (((MH “Sleep+”) OR (MH “Sleep Disorders, Circadian Rhythm”) OR (MH “Dyssomnias+”) OR (MH “Sleep Disorders+”) OR (MH “Sleep Arousal Disorders”) OR (MH “Sleep-Wake Transition Disorders+”) OR (MH “Jet Lag Syndrome”)) OR AB sleep OR AB insomnia OR AB sleep initiation and maintenance disorders OR AB sleep disorders, circadian rhythm OR AB sleep disorder*) AND (AB guideline OR AB consensus OR AB systematic review OR AB meta OR AB evidence summary OR AB recommend OR practice).

### Literature inclusion and exclusion criteria

The inclusion criteria were as follows: (i) shift workers or healthy participants in simulated shift work scenarios (including shift workers in healthcare, transportation, public service, production, and manufacturing), including night-shift workers; (ii) the study focused on the non-pharmacological management of sleep disorders associated with shift work and provided clear recommendations or interventions; (iii) the literature types included guidelines, expert consensuses, systematic reviews, meta-analyses, evidence summaries, clinical decisions, and best practices; and (iv) the literature was published in Chinese or English.

The exclusion criteria were as follows: (i) incomplete literature information, including research proposals, drafts, reports, or abstracts; (ii) the full text was not available; and (iii) literature that failed the quality evaluation.

### Literature quality evaluation criteria

The quality evaluation criteria according to the type of literature are as follows: (i) the guidelines were evaluated with the “Appraisal of Guidelines for Research and Evaluation II (AGREE II)” [14]; (ii) expert consensuses, meta-analyses, and systematic reviews were evaluated with the corresponding evaluation criteria of JBI Evidence-based Health Care Centers in Australia (2016 Edition) [13]; (iii) clinical decisions, recommended practices, and evidence summaries are types of thematic evidence summaries in the “6S” pyramid model of evidence-based resources. Since the evidence development process for these summaries is similar, their quality was assessed using the “Critical Appraisal for Summaries of Evidence checklist” [15].

### Literature quality evaluation process

The literature quality evaluation process was completed independently by two researchers who have systematically studied evidence-based nursing. In the case of a disagreement, the final decision was made by a third person with evidence-based research training experience. If there were any conflicts about the conclusions drawn from different sources of evidence, this study followed the principle of evidence priority, high-quality evidence priority, and the latest published authoritative literature priority.

### Criteria for determining the evidence and recommendation levels

The included evidence was graded and recommended according to The Australian JBI Evidence-based Health Care Centre Evidence Recommendation Level System (2014 Edition) [13]. The level of the evidence was divided into levels 1 to 5 according to the design category of the included study, Level 1 (randomized controlled trial), Level 2 (quasi-randomized controlled trial), Level 3 (observational—analytical study), Level 4 (observational—descriptive study), and Level 5 (expert opinion/basic study). The evidence was classified into grade A (strong recommendation) and grade B (weak recommendation) based on the JBI feasibility, appropriateness, meaningfulness, and effectiveness (FAME) structure.

## Results

### General characteristics of the included articles

**Fig. 1:**
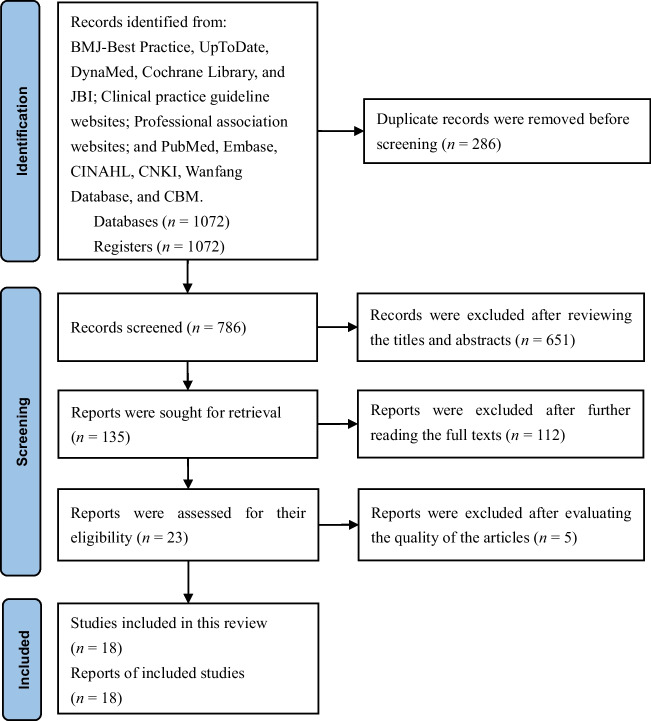
Screening flow chart for the articles

This study initially obtained 1072 articles. After removing 286 duplicate articles through Endnote checking, 651 articles were excluded after reviewing the titles and abstracts. After further reading the full texts, an additional 112 articles were excluded, and 23 articles were retained. After evaluating the quality of the articles, 18 articles were ultimately retained, including two clinical decisions [16, 17], two guidelines [18, 19], three expert consensuses [20–22], and 11 systematic reviews [11, 12, 23–31]. The screening flow chart for the articles is shown in Fig. 1. The general characteristics of the included articles are shown in Table 1.

**Table 1:** General characteristics of the included articles (*n* = 18)

Included articles	Topic	Source	Type of evidence	Year
Cheng et al. [16]	Sleep-wake disturbances in shift workers	UpToDate	Clinical decision	2022
Hilbert [17]	Shift work disorder	DynaMed	Clinical decision	2022
Patterson et al. [18]	Evidence-based guidelines for fatigue risk management in emergency medical services	PubMed	Guideline	2018
Smith et al. [19]	Use of actigraphy for the evaluation of sleep disorders	American Academy of Sleep Medicine	Guideline	2018
Wong et al. [20]	A multi-level approach to managing occupational sleep-related fatigue	Work Time Society	Expert consensus	2019
Ritonja et al. [21]	Individual differences in shift work tolerance and recommendations for research and practice	Work Time Society	Expert consensus	2019
Watson et al. [22]	Recommended amount of sleep for a healthy adult	PubMed	Expert consensus	2015
Reynolds et al. [23]	Is cognitive behavioral therapy for insomnia (CBTi) efficacious for treating insomnia symptoms in shift workers?	Embase	Systematic review	2023
Lam et al. [12]	Dose-response effects of light therapy on sleepiness and circadian phase shift in shift workers?	Embase	Systematic review	2021
Patterson et al. [24]	Does the evidence support naps on the night shift?	CINAHL	Systematic review	2021
Robbins et al. [25]	Workplace-based employee health interventions and their impact on sleep duration among shift workers	CINAHL	Systematic review	2021
Kang et al. [26]	Sleep quality among shift-work nurses	PubMed	Systematic review	2020
Shriane et al. [11]	Sleep hygiene in shift workers	Embase	Systematic review	2020
Patterson et al. [27]	Does evidence support “banking/extending sleep” by shift workers to mitigate fatigue, and/or to improve health?	Embase	Systematic review	2019
Temple et al. [28]	The effects of caffeine in fatigued shift workers	CINAHL	Systematic review	2018
Barger et al. [29]	Effect of fatigue training on safety, fatigue, and sleep in shift workers	CINAHL	Systematic review	2018
Martin-Gill et al. [30]	Effects of napping during shift work on sleepiness and performance in shift workers	CINAHL	Systematic review	2018
Patterson et al. [31]	Shorter versus longer shift durations to mitigate fatigue and fatigue-related risks in shift workers	PubMed	Systematic review	2018

### Quality evaluation results of the included articles

Quality evaluation results of the clinical decisions: This study included two evidence summaries, both of which were clinical decisions [16, 17]. The study by Cheng et al. [16] was scored “partially” for item 3, “clear and transparent review,” item 4, “comprehensive and transparent search,” and item 7, “appropriate citation of recommendations.” In contrast, item 5, “clear evidence grading,” was scored “no.” All the other items were scored as “yes.” The study by Hilbert [17] was scored “partially” for item 4, “comprehensive and transparent search,” and item 5, “clear evidence grading,” while all the other items were scored as “yes.” The overall process of developing the clinical decisions was rigorous, and the content was detailed; thus, they were included.

Quality evaluation results of the guidelines: This study included two guidelines [18, 19]. The percentage of standardization in each field and the average score of the two comprehensive evaluations are shown in Table 2.

**Table 2:** AGREEI II scores of the included guidelines (*n* = 2)

Included guidelines	Percentage of field standardization (%)						≥60% field number	≥30% field number	Recommendation level
	Scopes and objects	Involved personnel	Rigor of the guidelines	Clarity of the guidelines	Application of the guidelines	Independence of the guide			
Patterson et al. [18]	91.67	91.67	89.58	97.22	66.67	75.00	6	6	A
Smith et al. [19]	94.44	91.67	82.29	97.22	77.08	66.67	6	6	A

Quality evaluation results of the expert consensuses: There were three expert consensuses included in this study [20–22]. For item 1, “Is the source of the opinion clearly identified?” The three expert consensuses [20–22] were evaluated as “no.” For item 6, “Is any incongruence with the literature/sources logically defended?” Two of the expert consensuses [20, 21] were evaluated as “no,” while one of the expert consensus [22] was evaluated as “yes.” The remaining items were evaluated as “yes” in the three expert consensuses [20–22].

Quality evaluation results of the systematic reviews: In this study, 11 systematic reviews [11, 12, 23–31] were included. For item 1, “Is the review question clearly and explicitly stated?” Three of the systematic reviews [11, 23, 25] were evaluated as “unclear.” For item 4, “Were the sources and resources used to search for studies adequate?” One of the systematic reviews [11] was evaluated as “no.” For item 8, “Were the methods used to combine studies appropriate?” One of the systematic reviews [11] was evaluated as “unclear.” For item 11, “Were the specific directives for new research appropriate?” Only one of the systematic reviews [26] was evaluated as “unclear.” The remaining systematic reviews and evaluation items were all evaluated as “yes.” The methodological quality of the included systematic reviews was generally good.

### Evidence summary and description

Through the evidence extraction, classification, and synthesis, 25 pieces of best evidence were summarized from six aspects: sleep assessment, sleep scheduling, sleep hygiene, light therapy, workplace intervention, and other managements (Table 3).

**Table 3:** Evidence summary on the non-pharmacological management of sleep disorders in shift workers

Evidence item	Evidence content	Evidence level	Recommendation level
Sleep assessment	Sleep diaries and actigraphy are recommended as primary objective assessment tools for shift-work sleep disorders [16, 17, 19].	5	B
Sleep scheduling	It is recommended to have a total sleep time of 7–9 h [22]. Daytime sleep can be separated into two periods to accommodate the need for flexibility. It is also recommended to prioritize a regular 3–4 h fixed sleep period in the morning, and the duration of the second sleep period can be adjusted as needed [16].	5	B
	Shift workers need at least 11 h between shifts to obtain 7 h of sleep [20].	5	B
	To the greatest extent possible, regularity should be maximized in both activity and sleep schedules. Minimizing discrepancies in sleep timing between on-shift and off-shift periods may help adapt a fixed-shift schedule and each shift type in a rotating-shift schedule [16, 17].	5	B
	“Banking Sleep” before a shift can improve work performance, reduce acute fatigue, and decrease sleep onset latency during the shift work period [27].	2	B
	It is recommended that scheduled naps (less than 1 h) before or during shift work can improve drowsiness or fatigue and increase alertness and performance [17, 18, 24, 30].	2	B
Sleep hygiene	Creating a sleep-friendly bedroom environment by making sure that the bed is comfortable and that the bedroom is dark, quiet, and cool (around 65 °F or 18 °C) and creating constant background noise with a fan or humidifier may help reduce awakenings caused by fluctuations in the environment and unexpected sounds [11, 16, 17].	5	A
	Avoiding caffeinated products, including coffee, tea, chocolate, and other carbonated beverages, within 6 h before bedtime can affect the ability to initiate and maintain sleep [11, 16, 17].	5	A
	It is not recommended to use alcohol to help sleep, and alcohol consumption should be avoided within 3 h before bedtime, as it can cause frequent awakenings and disrupt sleep [11, 16, 17].	2	A
	Activities that raise body temperatures, such as exercise or a warm bath, should be avoided within 1.5 h of bedtime since they can disrupt the ability to fall asleep [11, 16, 17].	5	A
	Limiting fluid intake before bedtime and emptying the bladder before sleep can help prevent awakenings since having a full or partially full bladder can cause awakenings [11, 16, 17].	5	A
	Avoiding smoking and other drugs before bedtime can affect the ability to initiate or maintain sleep, as cravings can interrupt sleep [11, 16, 17].	5	A
	Activities that provide mental stimulation, such as watching TV or using electronic devices, should be avoided within 1 h before bedtime since electronic devices can mimic sunlight and limit sleep onset. Instead, relaxing and enjoyable activities such as reading or meditation are recommended [11, 16, 17].	5	A
Light therapy	Short-term exposure to moderate-intensity light (1000–5000 lux) during a night shift can reduce drowsiness, while exposure to higher-intensity light (>5000 lux) can regulate the phase shift of the circadian rhythms [12].	1	B
	Limiting exposure to bright lights before going to bed during the day can help promote sleep. Wearing blue-light filter glasses, dark goggles, or sunglasses can filter out some of the bright lights and help adjust the circadian rhythms [11, 16, 17].	5	A
Workplace intervention	It is recommended to develop an “optimal” work schedule to provide adequate opportunities for sleep, which requires the consideration of many factors such as hours of work, rest breaks between and within shifts, shift sequences, work demands, and workforce demographics and culture [20].	5	B
	It is not recommended to have a shift lasting more than 24 h [18, 31]. At least two night-sleep periods with a day off between shifts are required to offset the accumulated work-related fatigue [20, 21].	5	B
	Short, frequent breaks (up to 30 min) may be more protective than fewer, long breaks [20].	5	B
	Interventions or programs such as fatigue training and sleep education are beneficial for shift workers [29]. It is recommended to organize projects related to sleep or fatigue education and training programs to help employees understand how to ensure adequate sleep between shifts and to reduce fatigue and fatigue-related risks [18, 20].	3	B
	During night shifts, caffeine can increase alertness and reduce acute fatigue, but caffeine can also reduce sleep time and quality [18, 28].	1	A
	Interventions based on the workplace, such as sleep hygiene, shift schedules, mindfulness and stress reduction, light therapy, and napping, can increase the sleep time of shift workers [25].	2	B
Other managements	It is recommended to exercise for at least 5 h per week to improve overall health and reduce related diseases and complications [17].	5	A
	It is recommended to involve family members in the sleep treatment plan, especially in adapting sleep schedules for shift workers [17].	5	A
	Aromatherapy inhalation therapy can reduce discomfort during sleep, induce sufficient deep sleep time, and positively improve sleep quality for shift workers [26].	2	B
	The existing cognitive-behavioral therapy for insomnia may not be entirely applicable to shift workers, and tailored approaches to CBTI are needed for shift workers to improve sleep efficacy [23].	1	B

*CBTI* cognitive behavioral therapy for insomnia

## Discussion

### Reasonably arrange sleep time and improve the regularity of the sleep time

The American Academy of Sleep Medicine [22] and the National Sleep Foundation [32] recommend that adults aged 18–64 should regularly obtain 7–9 h of sleep each night to promote optimal health. However, for shift workers, at least 9–11 h of sleep time between shifts are required to obtain the 7 h of sleep needed to maintain health [20] and reduce the risk of impaired performance, increased errors, and injuries or accidents. Shift workers often must change social and family obligations. It is recommended that shift workers choose a work schedule that aligns with their sleep preferences to adapt to their needs [16, 17]. It is also recommended to prioritize 3–4 h of regular fixed sleep in the morning; the second sleep time can be adjusted as needed. Recent evidence suggests that adequate worker “control” over working hours (e.g., allows workers to adapt their working time arrangements to their personal needs can be a significant protective factor in reducing work-life or work-family conflict [6]. Evidence has suggested that “banking sleep” before shifts can improve work performance, acute fatigue, and sleep latency during the shift [27]. However, there is currently a lack of a standard definition or description for “banking sleep,” and some studies have defined “banking sleep” as a period of 8–10 h in bed [27]. Future research should establish a relative standard definition for “banking sleep” to clarify whether it refers to sleep time or all sleep obtained within 24 h, including napping.

Evidence has suggested that arranging naps before or during a shift can improve drowsiness or fatigue and enhance alertness and performance [17, 18, 24, 30]. Brief naps (≤ 30 min) can improve drowsiness and fatigue while reducing the risk of impaired performance. After a long nap (> 120 min), immediate performance may be affected, but performance can improve after 1 h [24]. Strategically arranging short naps while ensuring the safe and effective performance of work duties and considering the effects of sleep inertia is recommended. In addition, shift workers are advised to maximize the regularity of their activities and sleep schedules, minimize the differences in sleep times between shifts and non-shifts to adapt to a fixed shift schedule and shift types, and promote the stability of their circadian rhythm [16, 17]. In summary, the sleep time and arrangement of shift workers should be personalized to ensure sufficient total sleep time and improve the regularity of their sleep time.

### Develop good sleep hygiene and cultivate high-quality sleep

Sleep hygiene refers to individual practices or routines designed to optimize sleep time and quality, including the management of environmental factors [11]. Sleep hygiene is widely recommended for treating sleep disorders, whether used as a standalone intervention or in conjunction with psychological and pharmacological interventions. Evidence has suggested that exposure to bright lights should be limited to during the day to prevent increased alertness levels of the circadian rhythm [11, 16, 17]. However, exposure to bright lights during the night shift can suppress melatonin secretion to counteract the disruption of the circadian rhythm in shift workers [12], thus increasing their total sleep time. In practice, Chinese nurses often turn off all the lights at the nurse station during the night to let the patients and their families near the unit have a good rest, and they continue to work in a dimly-lit environment, which is not conducive to improving their sleep after shift. Studies have also shown that shift workers often change their bedroom environments to optimize sleep. It has been reported that 70% of nurses try to reduce noise levels [33], and 81% of shift workers from different backgrounds limit the lighting in their bedrooms [11]. Despite actively controlling their environmental factors such as temperature, lighting, and noise, some shift workers engage in stimulating activities before bedtime, including watching TV or playing video games in bed [34]. This could be explained by providing them with an opportunity to relax; however, the light emitted from electronic devices can mimic sunlight, delaying the onset of and disrupting their sleep. Therefore, engaging in relaxing and enjoyable activities such as reading or meditation 1 h before bedtime is recommended.

Furthermore, caffeine is a recognized fatigue management strategy that can increase alertness and performance [18, 28]. Shift workers commonly report using caffeine, but it can negatively affect the ability to initiate sleep while shortening sleep duration. Considering that caffeine has a half-life of 6–8 h, it is advised to avoid caffeine-containing products within 6 h before bedtime [11, 16, 17]. Shift workers also commonly report using alcohol to induce sleepiness, but alcohol can cause frequent awakenings, affecting restorative sleep [4]. Therefore, it is not recommended to use alcohol to facilitate sleep or to drink alcohol within 3 h before bedtime [11, 16, 17]. Shift workers adhere to sleep hygiene recommendations to varying degrees, but overall, they receive limited attention. Findings show that shift workers frequently report caffeine consumption and daytime napping, in line with best-practice fatigue-management strategies, but contrary to existing sleep hygiene recommendations, further research is required. Specifically, assessment of the applicability of current sleep hygiene guidelines to shift workers (particularly caffeine and napping recommendations) is required [11]. The results of this study can be used for the education and training of shift workers in sleep hygiene, even during non-shift periods, to manage work-related fatigue and cultivate high-quality sleep.

### Implementing a workplace intervention and promoting good sleep

Improving sleep and reducing sleep deprivation and fatigue are a priority for shift workers, particularly those who work in high-risk environments. Existing evidence has suggested that workplace interventions can improve sleep and sleep-related outcomes, including public safety and worker productivity [25]. Although the strength of the evidence is relatively weak, individual-focused interventions are unlikely to be effective without strong support at the workplace and organizational levels. In addition, existing evidence has suggested that organizations should develop “optimal” work schedules that provide employees with adequate sleep and recovery opportunities. Factors to consider when developing work schedules include hours of work, rest breaks between and within shifts, shift sequences, work demands, and workforce demographics and culture [20]. From a preventive point of view, work scheduling should be targeted as an intervention strategy on the workplace or group level to promote better sleep and minimize social impairments. The following recommendations for working time arrangements can be included: work scheduling should aim at regular, predictable work hours [6]. Shifts should not exceed 24 h [18, 31], and workers should be provided with at least two nights of sleep between shifts and 1 day of rest between shift cycles [20, 21]. Studies have shown that if shift work is involved, fast rotating shifts that allow for at least some work-free evenings each week are also a way to minimize social impairments in shift workers. If it is impossible to redesign shift schedules, training and educating employees and their families to balance their work and life can be beneficial [6]. Furthermore, workplace education and training programs on sleep and fatigue are recommended [18, 20]. Key program content can include basic information on sleep and circadian rhythms, sleep disorders, fatigue countermeasures such as caffeine and nap strategies, optimizing schedules or sleep environments, mindfulness, and good health behaviors. In addition to actions aimed at adjusting working hours and training and education, providing sufficient child care and support for workers (and their families) engaged in shift work can reduce stress among parents who work shifts and benefit children’s development [6]. There are also some promising sleep health promotion interventions aimed at modifying the workplace environment [35], although they were not included in this study due to the strength of the evidence. The characteristics of the shift worker population, such as age, gender, geographic location, and social and cultural factors, widely vary and may affect the effectiveness of specific sleep interventions and target sleep features such as duration, timing, and quality. Future research is necessary to identify more effective interventions for specific shift workers in their workplaces.

## Strengths and limitations

Our study has numerous strengths. First, we used a list of comprehensive search terms developed by in-depth search and discussions among the review team, and the search was run on a wide range of databases and then evaluated, classified, and summarized the evidence by using the method of evidence-based nursing, and finally formed the best evidence. Second, previous studies have focused on different interventions for the management of sleep disorders in shift workers, and this study systematically synthesizes the high-quality evidence on this topic, and the results should be of interest to shift workers or work organizations with sleep disorders. Third, we contacted the authors of some included studies to obtain additional data when they had not been reported in the published studies. Unfortunately, we did not receive feedback from all the authors. Finally, the risk of bias in the majority of the included studies was low.

Meanwhile, several limitations need to be acknowledged. First, this evidence summary only includes published studies in Chinese and English, and articles in other languages could be included to form a better evidence summary. Second, although we tried to make the literature search as exhaustive as possible, some original studies might have been missed, which the risk of publication bias might have remained. Third, as most evidence comes from expert opinions and there are few pieces of evidence derived from RCTs, 14 out of 25 pieces of evidence had grade “Level B - weak recommendation.” It is advisable for readers to carefully and critically select the best available evidence. At the same time, the best evidence summary will change continuously over time, and researchers should continuously update it.

Overall, although the evidence summarized in this study provides some reference for sleep management in shift workers, the type of work, the work schedules and their interrelation, and the intensity of work by shift-work period can also affect the sleep time and quality of shift workers, and this study did not give recommendations for shift workers in different industries, mainly due to the limited quality and quantity of relevant original studies or secondary studies at present.

Therefore, it is necessary to carry out high-quality original or secondary studies by taking different shift work groups as examples in the future to enrich the specific evidence in this area.

## Conclusion

Based on the evidence-based “6S” pyramid model, this study integrated the best evidence for the non-pharmacological management of sleep disorders in shift workers through evidence-based nursing, involving six aspects: sleep assessment, sleep scheduling, sleep hygiene, light therapy, workplace intervention, and other managements. This study provides guidance and acts as a reference for sleep problems and related education and training of shift workers, aiming to improve their sleep and sleep-related outcomes, including health, productivity, absenteeism, and other sleep-related problems. Additionally, although the best evidence was developed through a rigorous evidence-based approach, some of the recommended opinions were based on expert opinions or lower levels of evidence. Therefore, it is necessary to conduct scientifically rigorous original research to provide higher quality evidence for the non-pharmacological management of sleep disorders in shift workers.

## Data Availability

The datasets generated and/or analyzed during the current study are available from the corresponding author on reasonable request.
